# Recent advances in gold nanostructure-based biosensors in detecting diabetes biomarkers

**DOI:** 10.3389/fbioe.2024.1446355

**Published:** 2024-09-17

**Authors:** Tahereh Jamshidnejad-Tosaramandani, Soheila Kashanian, Kobra Omidfar, Helgi B. Schiöth

**Affiliations:** ^1^ Nanobiotechnology Department, Faculty of Innovative Science and Technology, Razi University, Kermanshah, Iran; ^2^ Department of Surgical Sciences, Division of Functional Pharmacology and Neuroscience, Uppsala University, Uppsala, Sweden; ^3^ Biosensor Research Center, Endocrinology and Metabolism Molecular–Cellular Sciences Institute, Tehran University of Medical Sciences, Tehran, Iran; ^4^ Sensor and Biosensor Research Center (SBRC), Faculty of Chemistry, Razi University, Kermanshah, Iran; ^5^ Endocrinology and Metabolism Research Center, Endocrinology and Metabolism Clinical Sciences Institute, Tehran University of Medical Sciences, Tehran, Iran

**Keywords:** diabetes mellitus, biosensor, gold nanostructures, biomarker, glucose detection

## Abstract

Diabetes mellitus (DM) is a prevalent disorder with an urgent need for continuous, precise, and on-site biomarker monitoring devices. The continuous monitoring of DM biomarkers from different biological matrices will become routine in the future, thanks to the promising biosensor design. Lately, employing different nanomaterials in biosensor receptor parts has had a great impact on smart DM monitoring. Among them, gold nanostructures (AuNSs) have arisen as highly potential materials in fabricating precise DM biosensors due to their unique properties. The present study provides an update on the applications of AuNSs in biosensors for detecting glucose as well as other DM biomarkers, such as glycated hemoglobin (HbA1c), glycated albumin (GA), insulin, insulin antibodies, uric acid, lactate, and glutamic acid decarboxylase antibodies (GADA), with a focus on the most important factors in biosensor performance such as sensitivity, selectivity, response time, and stability. Specified values of limit of detection (LOD), linear concentrations, reproducibility%, recovery%, and assay time were used to compare studies. In conclusion, AuNSs, owing to the wide electrochemical potential window and low electrical resistivity, are valuable tools in biosensor design, alongside other biological reagents and/or nanomaterials.

## Highlights

1. AuNSs presented outstanding properties such as chemical stability, high electrical conductivity, a large specific surface area, low cost of synthesis, and ease of functionalization.
2. The utilization of AuNSs in biosensors is an excellent opportunity to detect all diabetes biomarkers, such as glucose, HbA1c, GA insulin, IAA, and GADA.
3. AuNSs can improve the efficiency, sensitivity, specificity, and stability of the diabetes biosensors.
4. AuNS properties can increase the efficiency, sensitivity, and specificity of the biosensor through intrinsic specific molecule recognition capacity, different signal transduction amplification methods, fast electron transfer, and the ability to stabilize GOx structures and/or antibodies over proper immobilization on electrodes, serving as fluorescent and colorimetric materials.
5. Further consideration is required to translate AuNS-based technologies into routine and functional biosensors, as the properties of AuNSs are dependent on their size, shape, and spatial arrangement.

## 1 Introduction

Diabetes mellitus (DM) is a chronic metabolic disorder recognized by persistently elevated blood glucose levels, commonly due to defects in insulin secretion, function, or absorption mechanisms (Association, 2014). It is the ninth leading cause of death globally, as reported by the World Health Organization (WHO) (Khan et al., 2020). The number of people with DM is predicted to increase by 700 million by the year 2045 worldwide because of the modern lifestyle (Saeedi et al., 2019). DM can lead to other long-term adverse health effects, including the escalated risk of cardiovascular disease (Damaskos et al., 2020), hypokalemia (Frier, 2014), nephropathy (Reutens and Atkins, 2011), retinopathy (Shah et al., 2021), blindness (Stratton et al., 2001), foot damage (Vas et al., 2018), falls (Macgilchrist et al., 2010), amputation (Khalil et al., 2023), cerebral edema (Durr et al., 1992), dementia (Pasquier et al., 2006), skin conditions (Wollina et al., 2020), and disability (Yoon and Kim, 2019) which profoundly affect patients’ quality of life (Jitendra et al., 2024). Although DM has no certain cure, continuous follow-up of the body’s glucose levels for appropriate management can minimize the disease complications and reduce its severity (Alam et al., 2021).

**FIGURE 1:**
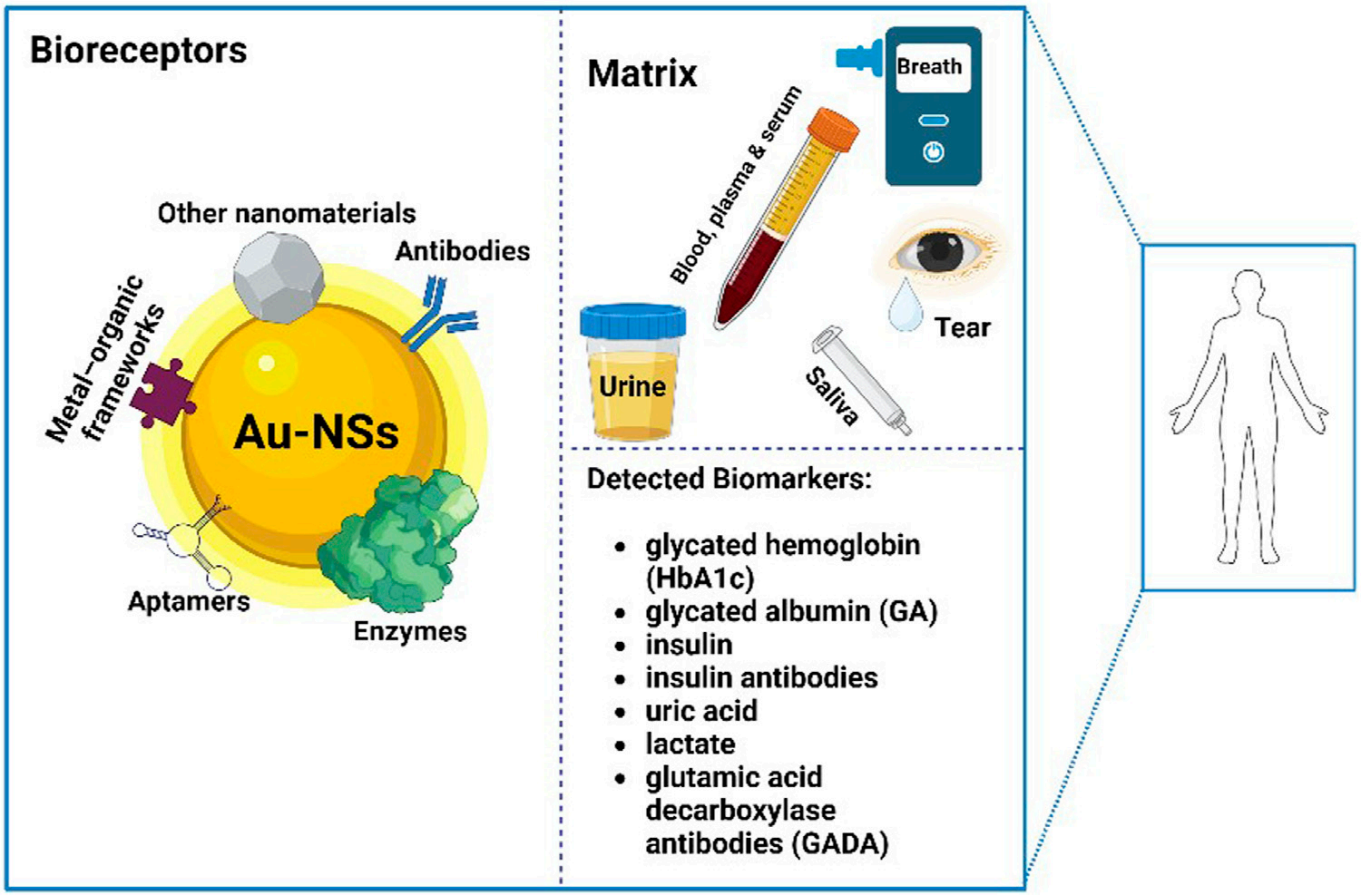
The principles of detecting diabetes mellitus biomarkers from different matrices in the human body using various bioreceptors and gold nanostructures.

The current standard of DM monitoring is through the invasive blood pricking technique for the detection of glucose (Kirk and Stegner, 2010). Typically, this method is reliable; however, the repetitive pricking in the long term is inconvenient for patients and can simply cause irritation and infections (Reddy et al., 2022). The current limitations of existing continuous monitoring biosensors are issues related to reliability, accessibility, complexity, cost, and time (Pullano et al., 2022). In contrast, the non-invasive, real-time, and continuous monitoring of glucose from different biological matrices (i.e., blood, urine, saliva, breath, interstitial fluids, tears, and sweat) has the potential to become routine in the near future (Laha et al., 2022). Furthermore, detecting other DM biomarkers such as insulin, insulin antibodies, HbA1c, GA, and acetone from different biological matrices using novel biosensor technology has remained an active subject of research (Reddy et al., 2022). This offers advantages such as low cost, the ability to detect low concentrations of biomarkers, and time and labor efficiency (Ahmadi et al., 2020; Laha et al., 2022; Wang et al., 2022; Jadhav et al., 2023; Mandali et al., 2023; Psoma and Kanthou, 2023). Different types of advanced point-of-care (POC) platforms make the monitoring of the DM biomarkers more precise, straightforward, safe, and less uncomfortable for patients (Teymourian et al., 2020; Liu Y. et al., 2022; Khor et al., 2022; Li and Chen, 2023; Safarkhani et al., 2023; Hu et al., 2024) (Figure 1).

Recently, advances in nanomaterials have prompted the designing of promising receptors for the development of cost-effective, on-site, and smart biosensors for DM monitoring (Shoaib et al., 2023). For instance, the surface area of the biosensors can be increased via nanomaterials, which results in generating greater current, more rapid reactions, and improved catalytic activity (Cho et al., 2020). Signal-to-noise ratio improvement is possible due to the amplifying properties of nanomaterials (Kumar et al., 2019). In addition, when designing multifunctional bioreceptors, different nanomaterials and/or biomolecules can be used in the biosensor simultaneously (Theyagarajan and Kim, 2023), or a susceptible biological substance can be replaced by mimicking materials entirely (Mohammadpour-Haratbar et al., 2022). These sensors are intended for the rapid detection of DM biomarkers with high selectivity, sensitivity, accuracy, and low detection limits (El-Safty and Shenashen, 2020; Welch et al., 2021). Last but not least, the upgrading of other biosensor properties such as stability, scalability, miniaturization, wearability, and connectivity to smart devices is possible now thanks to nanotechnology advances (Altintas, 2017; Golsanamlou et al., 2023; Mansour et al., 2024). Different kinds of nanomaterials applied in the construction of DM biosensors, including silver, nickel, carbon-based, and quantum dots, bring their own advantages based on their applications and properties (Malik et al., 2023). Depending on the specific requirements of the biosensors, such as desired sensitivity, stability, cost, and time, gold nanostructures (AuNSs) show great potential in this regard (Dahan et al., 2023).

Accordingly, in recent years, progress has been made in AuNS applications in the construction of biosensors (Naresh and Lee, 2021). More specifically, the AuNSs have emerged as potent tools in the fabrication of highly sensitive DM biosensors (Siciliano et al., 2023). Utilizing gold alongside other nanomaterials or biomolecules, such as enzymes and antibodies, to modify electrodes and fabricate AuNS-based biosensors can lead to improved sensitivity and lower LOD compared to different types of biosensors (Xu et al., 2023). The well-known properties of gold, such as the relatively simple synthesis methods of AuNSs, the ability to serve as an effective nanomaterial for biomolecule immobilization, and integration with other nanomaterials to fabricate effective biosensors with an increased load of biomolecules per unit mass of particles, make it a promising choice for biosensor applications (Oliveira et al., 2023; Patil et al., 2023; Zdarta et al., 2023). Additionally, the potential of AuNSs in electron transfer between the electrode surface and the biomolecules highlights the remarkable applications of AuNSs in DM biosensor construction (Deepa et al., 2023).

Some minor drawbacks need to be taken into consideration while designing a DM biosensor based on AuNSs, such as the tendency of the AuNSs to aggregate as well as the detachment or degradation of the surface attachments due to the potential non-specific interactions with biomolecules, oxidation, leaching, and/or corrosion (Demir et al., 2024). These might happen over changes in sample matrix conditions, such as pH, temperature, light, and chemical interactions, which can lead to a loss of AuNS properties and cause potential toxicity (Ngernpimai et al., 2024a). Addressing these challenges requires careful design and optimization of AuNSs, including using stabilizing agents, optimizing surface chemistry, and implementing strategies to minimize environmental conditions (Ngernpimai et al., 2024b). Additionally, a comprehensive study of the impact of relevant conditions is essential to ensure the long-term stability, reliability, and biocompatibility of AuNS-based DM biosensors.

In this regard, Yi et al. reviewed the advances in gold nanomaterial-implemented wearable sensors in general healthcare-related applications with a focus on the gold nanomaterial fabrication method, the working mechanism, and the performance of electrochemical sensors, humidity/gas sensors, strain/pressure sensors, and colorimetric sensors (Yi and Xianyu, 2022). More recently, Patra et al. reviewed previous studies on the applications of Au nanocomposites in enzymatic and non-enzymatic glucose sensing mechanisms (Patra et al., 2024). Several methods of immobilizing glucose oxidase (GOx) on gold nanoparticles (AuNPs) for electrochemical glucose biosensors were described by Lipińska et al. (2021). However, an objective review of AuNS applications in biosensors for all forms of DM biomarker monitoring is needed. Here, we provide an update on the applications of AuNSs in DM monitoring biosensors with a focus on the key factors in real-life biosensor performance. These factors are conductivity potential, linearity, sensitivity, response time, and selectivity when analyzing the DM biomarkers in different biofluid samples. The types of AuNS-based biosensors reviewed here, on the basis of the transduction method, are electrochemical, optical, chemiluminescence, and calorimetric detection in the second and third generation of glucose biosensors. Fourth-generation sensors are not addressed. Other diabetes biomarkers, such as HbA1c, GA, insulin, insulin antibodies, uric acid, lactate, and GADA, are discussed in detail.

## 2 Gold nanostructure (AuNS) applications in glucose detection

Along with the development of several glucose detection techniques and biosensors, novel practical AuNSs continue to emerge in the non-invasive skin biosensing platforms (Pour et al., 2023). In this regard, Dervisevic et al. proposed a glucose-sensing skin patch applying a high-density silicon micropillar array (MPA) for electrochemically monitoring glucose levels in human sweat (Dervisevic et al., 2021). In their study, the working electrode was modified by depositing a Prussian blue (PB) layer and gold nanoparticle clusters embedded in chitosan (Ch-AuNP) to increase the active surface area, followed by the immobilization of GOx (Dervisevic et al., 2021). The role of the jointed MPAs was physical protection of the immobilized GOx (Dervisevic et al., 2021), which made it possible to detect the glucose from perspiration effectively (Dervisevic et al., 2021). Another wearable biosensing platform was designed by combining a three-dimensional hierarchical porous Au hydrogel-enzyme electrode with soft-MEMS technologies, using GOx with good durability over 15 days and a suitable selectivity (Li et al., 2021). Interestingly, the same study showed that with the assistance of a wireless or a methylene blue Bluetooth module, this wearable sensing platform achieved real-time and non-invasive glucose monitoring on human skin as well (Li et al., 2021). Similarly, continuous lactic acid monitoring was accomplished using lactate oxidase immobilized on the same sensing platform, further verifying the universality of the designed sensing platform (Li et al., 2021). Frajpour et al. developed two high-performance glucose biosensors based on the immobilization of GOx on PB-modified TiO_2_ nanotube arrays functionalized by Au and AgO NPs, which exhibited satisfactory sensitivity toward glucose (Farajpour et al., 2020). They used AuNPs because of their excellent conductivity, simplicity of fabrication, and cost efficiency (Farajpour et al., 2020). Another PB-based sensing platform was established via the cross-linking enzyme aggregates method (GOx_EA_@PB/Au/CC) (Yan et al., 2021). The coral-like gold micro/nanostructures were formed onto carbon cloth, followed by a PB electrochemical deposition to construct an electrochemical biosensor to detect both H_2_O_2_ and glucose (Yan et al., 2021) (see Table 1).

**TABLE 1:** Glucose biosensors based on Au-nanostructures.

Biosensor technology	Glucose bioreceptor	Matrix/treatments	Linear concentration range	LOD	Assay time	Reproducibility (RSD^a^)	Recovery (%)	Ref
Electrochemical	Au−Si-MPA/PB/Ch-AuNP-GOx^b^	0.1 M PBS and artificial sweat/untreated	50 μM–1.4 mM	26 ± 5 μM	30 min	∼5.3%	NR^c^	Dervisevic et al. (2021)
Electrochemical	GOx/Au hydrogel	0.1 M PBS and sweat at pH 7.4 & 4.4/untreated	0–5 mM	17.80 μM	Real-time	0.30%–0.70%	NR	Li et al. (2021)
Cyclic voltammetry (CV) and chronoamperometry	GOx/PB/Au modified-TiO_2_ NTs^d^	PBS^e^, pH 7.4/untreated	0.1–0.4 mM	4.91 μM	NR	NR	NR	Farajpour et al. (2020)
Chronocoulometric and CV	GOx@PB/Au/CC^f^	Serum/diluted 10 times with PBS, pH 6.0	0.05–3.15 mM	10 μM	NR	3.3%–4.3%	98%–104%	Yan et al. (2021)
SERS^g^	Au@Ag NPs	Blood/centrifuged and 10 mM of the HEPES solution were added	10^−1^–10^−6^ M	10–6 M	5 min	NR	NR	Pan et al. (2021)
SERS	Gold nanoparticles (AuNPs) and two-dimensional MXene Ti_3_C_2_TX nanosheets	Tear/untreated	1–50 µM	0.39 µM	10 s	11.7%	NR	Cui et al. (2022)
Amperometric	GOx/AuNPs/Pty/PB/SPCE^h^	Plasma/40-fold dilution with PBS, pH 6.0	1.0 μM–1.0 mM	1.0 μM	1.5 min	1.9%–4.3%	82.5 ± 3.5 to 100.3 ± 0.4	Khumngern et al. (2021)
Amperometric	Nf-GOx/PB/AuNPs/GR^i^	Serum/10-fold dilution with PBS 0.05 M, pH 7.4	0.025–1 mM	0.0088 mM	NR	0.69%–0.84% (n = 3)	101–102	Sakalauskiene et al. (2023)
EBFC^j^	CNTs/AuNPs/GOx^k^	Urine/untreated, pH 5.3, 5.9, 7.1, and 8	0.2–5 mM	NR	Real-time	3.7%	NR	Zhang et al. (2021)
NFC^l^ potentiostat	GOx-AuNPs-PEDOT:PSS/PB-G/SPCE^m^	Serum/100-diluted with 0.10 M PBS, pH 7.00	0.5–500 μM	0.15 μM	Real-time	No significant difference 0.68% (n = 6)	96 ± 2 to 104 ± 3	Promsuwan et al. (2023)
Fluorescence	BSA-AuNCs@-GOx^n^	Serum and urine/100 and 10 times diluted with water, respectively	25–225 mM	0.03 mM	Real-time	NR	94–101	Abraham et al. (2024)
Electrochemical	PbS CQDs/AuNSs/GOx^o^	PBS at pH 7.4	0.1 μM–10 mM	1.432 nM	NR	NR	NR	Zhao et al. (2023)
Colorimetric	EM-GOx-GNPs^p^	PBS and serum/untreated	0–15 mM	0.6 mM	NR	NR	NR	Jang et al. (2022)

^a^ RSD, Relative standard deviation

^b^ Au−Si-MPA/PB/Ch-AuNP-GOx, High-density silicon micropillar array/Prussian blue layer and gold-nanoparticle clusters embedded in chitosan/glucose oxidase

^c^ NR, Not reported

^d^ GOx/PB/Au modified-TiO_2_ NTs, immobilized GOx onto PB-modified TiO_2_ nanotube arrays functionalized by Au and AgO NPs

^e^ PBS, Phosphate-buffered saline

^f^ GOx@PB/Au/CC, coral-like gold micro/nanostructures on carbon cloth/PB

^g^ SERS, Surface-enhanced Raman scattering

^h^ GOx/AuNPs/Pty/PB, GOx on gold nanoparticles (AuNPs) with the adsorption on a polytyramine layer (AuNPs/Pty), coated on a Prussian blue (PB)-modified screen-printed carbon electrode (SPCE)

^i^ Nf-GOx/PB/AuNPs/GR, Nafion/graphite rod electrode modified by AuNPs and PB with GOx

^j^ EBFC, Enzymatic biofuel cell

^k^ CNTs/AuNPs/GOx, Carbon nanotubes/AuNP hybrids/GOx

^l^ NFC, Near-field communication

^m^ GOx-AuNPs-PEDOT, PSS/PB-G/SPCE: SPCE modified with PB-graphene ink and functionalized with AuNP-embedded poly (3,4 ethylene dioxythiophene), polysulfonic acid coated with GOx

^n^ BSA-AuNCs@-GOx, bovine serum albumin stabilized gold nanocluster modified with GOx

^o^ PbS CQDs/AuNSs/GOx, PbS colloidal quantum dots/Au nanospheres, 16EM-GOx-GNPs

In addition to electrochemical wearable glucose biosensors, research has been focusing on developing technologies to enable surface-enhanced Raman scattering (SERS) biosensors (Pan et al., 2021). For instance, Pan et al. reported a two-step seed-mediated synthesis of gold core@silver shell nanoparticles (Au@Ag NPs) for detecting diabetes over the etching effect of H_2_O_2_ generated from glucose oxidation (Pan et al., 2021). They used Au@Ag NPs as SERS substrates and 4-mercaptobenzoic acid (4-MBA) as the Raman tag to detect glucose concentration (Pan et al., 2021). The role of Au@Ag NPs was to improve the electromagnetic field of SERS owing to their strong plasmonic properties (Pan et al., 2021). Another flexible SERS substrate composed of AuNPs and two-dimensional MXene Ti_3_C_2_TX nanosheets has been designed to detect tear glucose (Cui et al., 2022). In the same way, when AuNPs were present, the combination of electromagnetic and chemical enhancement of AuNPs and MXene greatly enhanced the SERS signal (Cui et al., 2022). The GMXeP SERS substrates were used to detect glucose conveniently from diabetic tears with the significant correlation between tear and blood glucose, suggesting that the designed system was suitable for non-invasive and sensitive detection of blood glucose (Cui et al., 2022) (see Table 1).

In another study, for amperometric detection of glucose, GOx was immobilized on AuNPs with the adsorption on a polytyramine layer (AuNPs/Pty) (Khumngern et al., 2021). Then, GOx/AuNP/Pty was coated on a PB-modified screen-printed carbon electrode (SPCE) to produce the GOx/AuNP/Pty/PB/SPCE biosensor (Khumngern et al., 2021). In the same study, the developed amperometric glucose biosensor response was measured through the reduction current of the PB mediator in a flow injection analysis system, displaying a low value for the Michaelis constant (Khumngern et al., 2021). The immobilization of GOx with high affinity was accomplished via AuNPs thanks to the thiol- and amino-functional groups of the enzyme (Khumngern et al., 2021). The immobilization via AuNPs improved the enzyme loading and sensor response without disturbing the enzymatic activity (Khumngern et al., 2021). In the same way, a graphite rod (GR) electrode was modified by AuNPs and PB with GOx to develop an amperometric glucose biosensor in another study, using Nafion (Nf) to produce an Nf-GOx/PB/AuNP/GR biosensor (Sakalauskiene et al., 2023). The AuNPs increased the electrochemically active surface area, improved the GOx immobilization, and yielded a 1.86-fold improvement in analytical signal strength (Sakalauskiene et al., 2023). This study showed AuNSs could be interesting nanomaterials in the interface construction of biosensors due to their low electrical resistivity, relatively wide electrochemical potential window, improved electrooxidation, and fast electron transfer that can enhance the sensitivity and stability of biosensors (Sakalauskiene et al., 2023) (see Table 1).

There has been a boom in developing novel analytic devices that are miniature, user-friendly, rapid, and reliable by combining biosensors with new technologies. Zhang et al. designed a biofuel cell-type sensor device consisting of a glucose biofuel cell, a power management system (PMS), and an indicator module to detect glucose in urine (Zhang et al., 2021). The enzymatic biofuel cells (EBFCs) were manufactured on a flexible substrate by screen printing technology (Zhang et al., 2021). The carbon nanotube (CNT)/hybrids were immobilized on the anode to promote the electron transfer between the active site of enzymes and the electrode surface to improve the output performance of EBFCs (Zhang et al., 2021). The PMS circuit enabled collecting the energy and drive of the light-emitting diode (LED) indicator, whose flash frequency was related to the urine glucose level (Zhang et al., 2021). The designed device was self-powered and held potential application prospects in wearable monitoring systems such as diapers (Zhang et al., 2021). Promsuwan et al. introduced a glucose biosensor that included a smartphone and a battery-less near-field communication (NFC) potentiostat connected to an SPCE modified with a PB-graphene ink and functionalized with AuNP-embedded poly (3,4 ethylene dioxythiophene): polysulfonic acid coated with GOx ((GOx)-AuNP-PEDOT:PSS/PB-G) for glucose detection using an amperometric method (Promsuwan et al., 2023). The PEDOT:PSS was a conductive gel for the entrapment of the GOx-AuNPs and the improvement of electron transfer to the PB-G mediator (Promsuwan et al., 2023). The PB-G was used as a redox mediator to electro-catalyze the reduction of H_2_O_2_, which was a byproduct of the GOx reaction (Promsuwan et al., 2023) (Table 1).

The photoluminescence properties of Au in an enzymatic fluorescent probe were developed for the selective detection of glucose using bovine serum albumin stabilized gold nanoclusters (BSA-AuNCs), modified with GOx (Abraham et al., 2024). The red fluorescence exhibited by the probe was quenched by the production of H_2_O_2_ on the addition of glucose via a static quenching mechanism, providing UV-visible absorption and fluorescence-lifetime-based glucose sensing (Abraham et al., 2024). In the mentioned study, the fluorescent enzymatic sensing probe served as an off-switch with the production of H_2_O_2_, resulting in the selective and sensitive detection of glucose (Abraham et al., 2024). In another work, Zhao et al. demonstrated a glucose electrochemical biosensor through the synergetic labeling strategy utilizing PbS colloidal quantum dots (CQDs) and Au nanospheres (AuNSs) (Zhao et al., 2023). The PbS CQD/AuNS/GOx mixture was immobilized on the carbon electrode surface via the one-step dip-coating method (Zhao et al., 2023). Colorimetric glucose sensors using enzyme-coronated AuNPs have been developed for high-throughput assays (Jang et al., 2022). To increase the selectivity and stability in detecting blood glucose, the biosensors were functionalized with an erythrocyte membrane, which facilitates the permeation of glucose, thanks to glucose-selective membrane proteins (Jang et al., 2022). The performance of the biosensor is represented in Table 1.

## 3 Au-nanostructure-based biosensors for other diabetes biomarkers

Glycated hemoglobin (HbA1c) is an established DM biomarker, according to the World Health Organization and the American Diabetes Association. HbA1c can be used for the practical long-term diagnosis of the disease in clinical practice as an alternative to glucose (Sherwani et al., 2016). In this regard, an electrochemical HbA1c biosensor with good efficiency was designed based on the electrochemical immune principle. The reproducibility and conductivity of the electrode are improved by depositing AuNPs on the surface of the screen-printed electrode (SPE) (Zhao et al., 2022). The experimental results showed a sensitivity of 0.0938 μA/μg·mL^−1^ (Zhao et al., 2022). The sensor delivered satisfactory repeatability, stability, and anti-interference performance (Zhao et al., 2022). Additionally, Boonprasert et al. developed multiwalled nanotubes incorporated with gold nanoparticles (POC-HbA1cMWCNTs/AuNPs), used as a routine POC for the detection of HbA1c (Boonprasert et al., 2023). They compared their developed biosensor to the standard HPLC method and showed the accuracy of the POC-HbA1cMWCNTs/AuNPs was 94.18% (Boonprasert et al., 2023). Likewise, a sandwich paper-based electrochemiluminescence (ECL) biosensor was developed using the zirconium metal–organic framework/Fe_3_O_4_ (trimethyl chitosan)/gold nanocluster (Zr-MOF/Fe_3_O_4_(TMC)/AuNCs) as a tracing tag to label anti-HbA1c monoclonal antibodies and used reduced graphene oxide (rGO) as an immobilization platform for the sensing element (Ahmadi et al., 2021). The fabricated immunosensor demonstrated a desirable assay performance for HbA1c (Ahmadi et al., 2021). Furthermore, a thiol-modified aptamer containing AuNPs bound to HbA1c with high affinity in whole blood samples was synthesized by Devi et al. to produce a stable aptasensor (Devi et al., 2023). The results showed that the thiol groups enhanced the stability of aptamers adsorbed on the surface of AuNPs effectively (Devi et al., 2023) (see Table 2).

**TABLE 2:** Biosensors for other diabetes biomarkers based on Au nanocomposites.

Sensor technology	Detected biomarkers	Bioreceptor	Matrix/treatments	Linear concentration range	LOD	Assay time	Reproducibility	Recovery	Ref.
Electrochemical	HbA1c^a^	AuNPs/SPE^b^	PBS at pH 7.4/untreated	20–200 μg/mL	15.5 μg/mL	NR^c^	NR	NR	Zhao et al. (2022)
Electrochemical	HbA1c	MWCNTs/AuNPs/SPCE^d^	Blood/five times diluted with PBS	0.186–2.044 g/dL	0.01 g/dL	Real-time	NR	NR	Boonprasert et al. (2023)
Electrochemiluminescence	HbA1c	Zr-MOF/Fe_3_O_4_(TMC)/AuNCs and rGO^e^ on a SPE	Blood/five times diluted in the red cell lysis buffer	2%–18%	0.072%	NR	≤4%	NR	Ahmadi et al. (2021)
Colorimetric	HbA1c	G@NPs^f^	Blood/100 times diluted with deionized water, pH 7.4	0.1 μM–100 μM	0.1 μM	7 min	<5.1%	94.0% and 95.4%	Devi et al. (2023)
Electrochemical	GA^g^	AuPt NPs/µSPE^h^	Blood/untreated, pH 7	0.1 nM to 500 mM	0.1 nM	NR	3.28	NR	Mahobiya et al. (2023)
Electrochemical immunosensor	Insulin	Au@Cu_5_Zn_8_/HPCNC/GCE^i^	Human serum/diluted with PBS, pH 7.4	DPV^j^: 0.000022–11 ng mL^−1^; and amperometry 0.000022–222 ng mL^−1^ ^a^	0.341 for DPV and 0.453 fg mL^−1^ for amperometry	NR	4.63%	NR	Sakthivel et al. (2022)
Electrochemical aptasensor	Insulin	AuNPs-Apt/LSGEs^k^	Blood/0.1 M Tris-HCl, pH 7.4	0.1 p.m. to 1 μM	22.7 fM	NR	1.80%	90.93%	Liu et al. (2022b)
Electrochemical	Insulin antibodies	Polyaniline and gold NPs	Plasma/diluted with a PBS, pH 7.4	0.001–1,000 ng mL^-1^	0.017 pg mL^−1^ and 0.034 pg mL^−1^ in DPV and square wave voltammetry	6 min	5.7%	99%–104%	Farrokhnia et al. (2022)
SERS^l^	GADA and IAA^m^	Silver–gold core-shell nanotags embedded with Raman probes	Human serum/untreated	0.01–100 ng mL^−^ ^a^	NR	NR	6.87% and 7.96%	NR	Wang et al. (2023)
Electrochemical	Glucose and insulin	SPCE-AuNPs-GluApt-MB and SPCE-AuNPs-InsApt-MB^n^	Saliva/adding 0.5% sodium dodecyl sulfate (SDS) to the collected sample followed by heating up to 70°C for 10 min, pH 7.4	0.1–50 mM and 0.05–15 nM	0.08 mM and 0.85 nM	Real-time	2.67% for glucose, 1.52% for insulin	95.1–104.1 for glucose, 92.0–98.8	Liu et al. (2022c)
Electrochemical	Glucose and lactate	AuNNs-PEGDE^o^	Sweat/untreated, pH 7.4	0–250 μM and 0–25 mM	7 μmol L^−1^ ^a^ and 54 μmol L^−1^ ^a^	real-time	2.9%–4.3% and 3.2%–4.7%	92.8%–108% and 98.7%–106%	Yu et al. (2021)
Luminescent	Uric acid, glucose, and alcohol	Uricase/aGG-AuNCs@PAH@MnO_2_ NSs^p^, GOx/HG-AuNCs@PAH@MnO_2_ NSs^q^ and ADH/aGG-AuNCs@PAH@MnO_2_ NSs^r^	Sweat/untreated	0–125 μM, 0–50 μM, and 1–15 mM	136 nM and 0–4 mM	3 min	NR	NR	Zhou et al. (2021)

^a^ HbA1c, glycated hemoglobin.

^b^ MWCNTs/AuNPs/SPE, screen-printed electrode.

^c^ SPCE, multiwalled nanotubes incorporated with gold nanoparticles on a screen-printed carbon electrode.

^d^ NR, not reported.

^e^ Zr-MOF/Fe3O4(TMC)/AuNCs, zirconium metal–organic framework/Fe_3_O_4_ (trimethyl chitosan)/gold nanocluster and reduced graphene oxide.

^f^ G@NPs, Thiol-modified aptamer oligonucleotides containing gold nanoparticles.

^g^ GA, glycated albumin.

^h^ Au-Pt NPs/µSPE, bi-metallic gold-platinum nanomaterial on a micro-screen-printed electrodes.

^i^ Au@Cu5Zn8/HPCNC/GCE, gold nanoparticle-adhered metal–organic framework-derived copper–zinc hollow porous carbon nanocubes on a glassy carbon electrode.

^j^DPV, differential pulse voltammetry.

^k^ AuNPs-Apt/LSGEs, gold nanoparticle-aptamer probes and laser-scribed graphene electrodes.

^l^ SERS, surface-enhanced Raman scattering.

^m^ GADA and IAA, glutamic acid decarboxylase antibodies, and insulin autoantibodies.

^n^ SPCE-AuNPs-GluApt-MB, and SPCE-AuNPs-InsApt-MB, gold nanoparticles decorated SPCE, terminated with redox probes methylene blue.

^o^ AuNNs-PEGDE, gold nanopine needles-poly (ethylene glycol) diglycidylether.

^p^ aGG-AuNCs@PAH@MnO_2_ NSs, aGG-Au nanoclusters-poly (allylamine hydrochloride) MnO_2_ nanosheets.

^q^ GOx/HG-AuNCs@PAH@MnO_2_ NSs, glucose oxidase-Au nanoclusters-poly (allylamine hydrochloride) MnO_2_ nanosheets.

^r^ DH/aGG-AuNCs@PAH@MnO_2_ NSs, DH/aGG-Au nanoclusters-poly (allylamine hydrochloride) MnO_2_ nanosheets.

However, HbA1c detection is not recommended for specific conditions such as pregnancy, chronic kidney disease, and hemoglobinopathies (Yazdanpanah et al., 2017). In such situations, glycated albumin (GA) can be used as an alternative DM biomarker without the interference of other health issues (Mahobiya et al., 2023). Accordingly, Mahobiya et al. measured the level of GA instead of HbA1c with microscreen-printed electrodes (μSPE) coated with bi-metallic gold-platinum (AuPt) nanomaterial with a synergistic effect (Mahobiya et al., 2023). The developed sensing platform showed an improved response compared to singular Pt nanoparticles (Mahobiya et al., 2023) (see Table 2).

Precise insulin detection is crucial for managing DM through regulated insulin dosage (Turner and Pickup, 1985). Common laboratory analytical methods for insulin detection are usually cost- and time-consuming and lack a real-time and continuous monitoring potential (Psoma and Kanthou, 2023). Therefore, research efforts are aiming toward insulin biosensors to offer a more accurate estimation of insulin (Psoma and Kanthou, 2023). Consequently, a simple sandwich-type electrochemical immunosensor was fabricated using AuNP-adhered metal–organic framework-derived copper–zinc hollow porous carbon nanocubes (Au@Cu_5_Zn_8_/HPCNC) and AuNP-deposited nitrogen-doped holey graphene (NHG) was used as a dual functional label and sensing platform (Sakthivel et al., 2022). Similarly, Liu et al. designed an electrochemical aptasensor to detect insulin using laser-scribed graphene electrodes (LSGEs) (Liu J. et al., 2022). The aptasensor was based on using Exonuclease I (Exo I) (Liu J. et al., 2022). The results showed using the aptamer, AuNPs, MB, and Exo I, the signal could be well-correlated to the concentrations of insulin (Liu J. et al., 2022) (see Table 2).

The presence of antibodies against insulin can be part of diagnosing people with type 1 diabetes (Katsarou et al., 2017). In this regard, an electrochemical biosensor for rapid detection of insulin antibodies was developed (Farrokhnia et al., 2022). The fabrication process was based on the optimized sequential electropolymerization of polyaniline and electrodeposition of AuNPs on the surface of the functionalized gold electrode (Farrokhnia et al., 2022). After immobilizing the insulin antigen and blocking with BSA, the biosensor was successfully used to determine different concentrations of insulin antibodies under optimal conditions (Farrokhnia et al., 2022). In addition, an SERS-based biosensor using polyvinylidene fluoride (PVDF) membranes as a flexible support for the detection of GADA and insulin autoantibodies (IAA) was developed (Wang et al., 2023). In the same study, two kinds of silver–gold core-shell nanotags embedded with Raman probes and attached with GADA or IAA were synthesized to capture the targets (GADA and IAA) (Wang et al., 2023). Results showed the probes sandwiched between silver and gold layers guaranteed spectral stability and reliability (Wang et al., 2023). Another electrochemical aptasensor on SPCE was developed for real-time detection of insulin and glucose in saliva (Liu S. et al., 2022). Two specific aptamers for insulin and glucose were fabricated on AuNP SPCEs to form the sensing platform that terminated with methylene blue redox probes (Liu S. et al., 2022) (See Table 2).

In another combinational work, a gold nanopine needle (AuNN)-programmed flexible sweat sensor was developed for real-time monitoring of glucose and lactate levels in human sweat (Yu et al., 2021). The AuNNs were grown on the flexible gold substrate by electrochemical deposition for signal amplification (Yu et al., 2021). The corresponding enzymes were immobilized on the chip via a cross-linker poly (ethylene glycol) diglycidylether (PEGDE) (Yu et al., 2021). Zhou et al. designed a luminescent wearable sweat tape (LWST) biosensor that can be attached to a smartphone (Zhou et al., 2021). It embedded multi-component nanoprobes onto microwell-patterned paper substrates of hollowed-out double-side tapes consisting of responsive luminophores, enzyme-loaded gold nanoclusters (AuNCs), which were wrapped by the switch and MnO_2_ nanosheets (Zhou et al., 2021). The responsive luminophores were constructed using three substitutable components: first, uricase, GOx, and alcohol dehydrogenase enzymes for molecular target recognition of uric acid, glucose, and alcohol, respectively (Zhou et al., 2021). Second, glutathione-protected AuNCs (yellow, red, and green) for luminescent signal output, and third, polycation PAH (poly (allylamine hydrochloride)) for integration (Zhou et al., 2021). MnO_2_ NSs as the switch could quench the emission of the AuNCs but be degraded by the reductive product of the incorporated enzymes (Zhou et al., 2021). The results showed the targeting analysts could be detected through a “turn-on” luminescence approach (Zhou et al., 2021) (see Table 2).

## 4 Challenges and drawbacks in the commercialization of AuNS-based DM biosensors

Commercializing AuNS-based DM biosensors involves navigating several hurdles, including regulatory challenges, manufacturing scalability, cost-effectiveness, and user adaptation (Kumar and Mahajan, 2024). Accordingly, proving the safety and biocompatibility of the biosensor requires comprehensive studies to ensure that AuNS-based biosensors are safe (Lu et al., 2023). Potential toxicity, long-term stability, and environmental impact need thorough evaluation (Lu et al., 2023). In this context, obtaining approval from regulatory authorities could be stringent and time-consuming (Marimuthu et al., 2024). It usually involves extensive preclinical and clinical testing, quality control, and documentation (Marimuthu et al., 2024). Additionally, defining exclusive and universal standardization protocols for testing and validating the performance, safety, and quality control of heterogeneous AuNS-based biosensors can complicate regulatory approval (Marimuthu et al., 2024). Furthermore, manufacturing with consistent quality and performance at a large scale is challenging (Kumalasari et al., 2024). Variability in AuNS size, shape, and surface functionalization can affect biosensor translation to large-scale production (Chakraborty et al., 2024). Moreover, integration with smart devices in a reproducible and scalable manner requires advanced manufacturing techniques and robust quality control processes, which can lead to high production costs (Ghobashy et al., 2024). To address this challenge, collaborative efforts are needed between researchers, manufacturers, and regulatory bodies to help streamline the development and approval process (Ghobashy et al., 2024). This includes advancements in integration, automation, chemical synthesis, and process optimization to reduce costs (Zou et al., 2024). In summary, while AuNS-based biosensors hold significant promise for improving diabetes management, addressing these hurdles is critical for successful commercialization. Through collaborative efforts, innovative manufacturing, and cost-reduction strategies, these challenges can be overcome (Kumar and Mahajan, 2024).

## 5 Future perspective and emerging trends in AuNS-based DM biosensors

**FIGURE 2:**
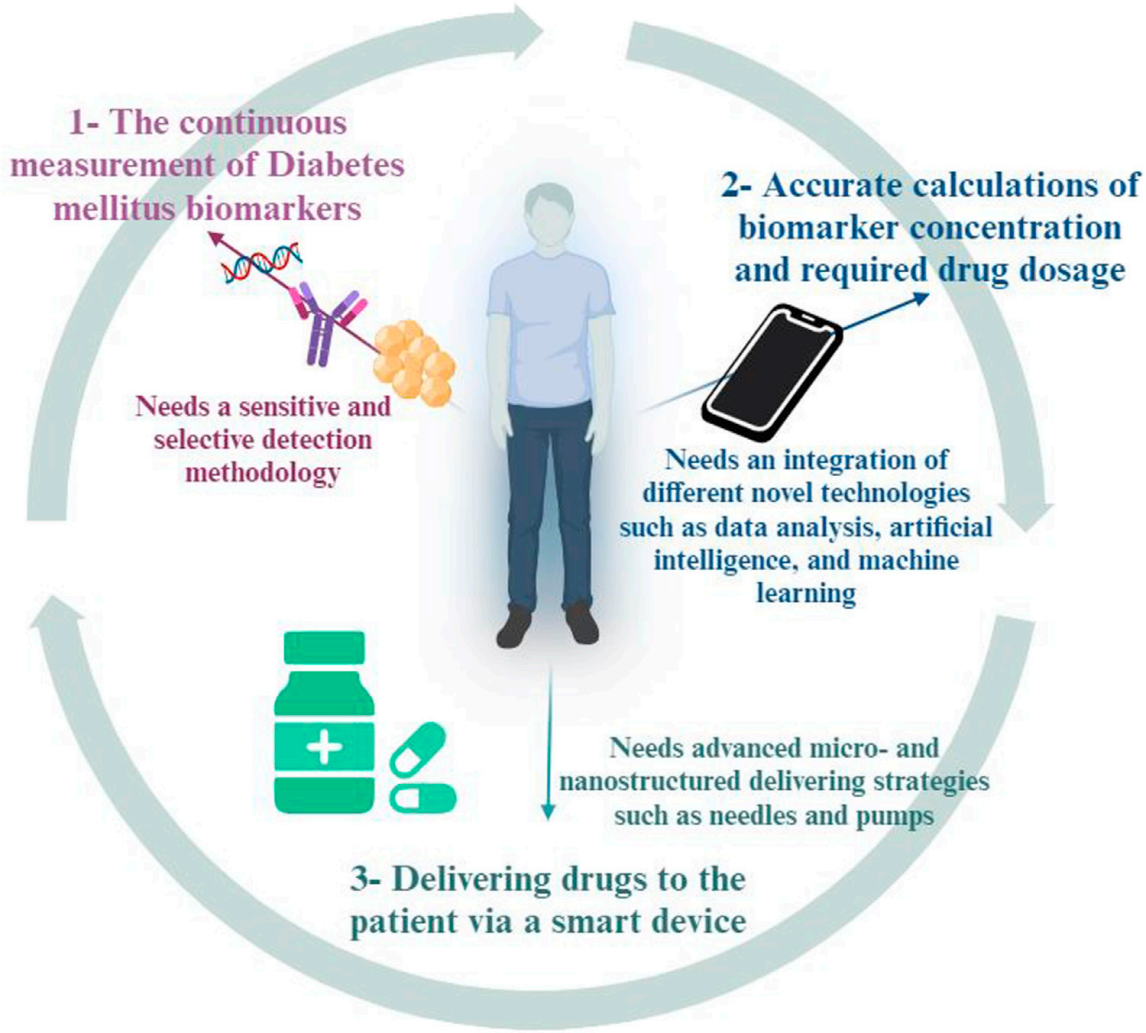
Closed loop structures can improve the overall management of diabetes mellitus (DM) by providing simultaneous biomarker detection and drug release.

Emerging trends in the application of AuNSs are one of the most successful examples of biosensor innovations for DM detection, offering exceptional biocompatibility, stability, and conductivity, paving the way for significant breakthroughs in POC (Haider et al.). Currently, AuNSs enhance the sensitivity and precision of glucose monitoring devices, enabling real-time, non-invasive glucose detection with higher accuracy (Arafa et al., 2024). Their ability to operate in various environmental conditions without the limitations associated with enzyme degradation makes them ideal for continuous glucose monitoring systems in non-enzymatic biosensors (Tehrani et al., 2024). Additionally, they enhance the sensitivity and specificity of biosensors for other DM biomarkers, facilitating real-time, non-invasive, and multi-detection DM monitoring through smart devices (Kim et al., 2024). As an emerging trend, the integration of AuNSs with advanced technologies like artificial intelligence and machine learning can improve data analysis and predictive capabilities (Ahmad and Muhmood, 2024; Darwish et al., 2024; Eswaran et al., 2024; Gupta et al., 2024; Zhou et al., 2024). Future perspectives also include the development of hybrid biosensors combining nanomaterials with other nano- and micro-structures to simultaneously monitor a broader range of biomarkers and the release of a drug in a closed loop to improve the overall management of DM (Wang et al., 2024) (Figure 2). This offers a more comprehensive health assessment and personalized treatment strategies in the future.

## 6 Discussion

This study aimed to shed light on the recent advances in Au-nanostructured-based biosensors in detecting glucose and other diabetes biomarkers, such as HbA1c, GA insulin, IAA, and GADA. This is imperative because DM has no certain cure to date, and a real-time measurement of the biomarkers for proper management of the disease can lower the risk of further complications. Previous studies showed that AuNSs exhibited excellent properties, including high electrical conductivity, a large specific surface area, relatively low cost of synthesis, and high biocompatibility. Thus, they are suitable options in the construction of biosensors for DM monitoring. AuNSs are valuable in biosensor construction due to their established low electrical resistivity and wide electrochemical potential window. In addition, they can enhance the sensitivity and stability of biosensors through properties such as intrinsic specific molecule recognition capacity, signal transduction amplification in different methods, fast electron transfer, and the ability to stabilize structures of GOx and/or antibodies on electrodes. They also can serve as fluorescent and colorimetric materials, benefiting from their excellent optical features based on an aggregation/dispersion switch.

The explanation for the wide applications of the Au-nanostructures in DM biosensor construction is their strong properties, such as chemical stability and consistent morphology and size, while detecting various biomarkers. Another important characteristic is the ease of functionalization of biosensors with biomaterials such as GOx and antibodies, which can increase the efficiency, sensitivity, and specificity of the biosensor. The high surface-to-volume ratio of the Au nanostructures allows for the attachment of many biomaterials and chemicals. Outstanding electrical conductivity enables a reliable signal response for various biomarkers in different biological matrices. Last but not least, good biocompatibility, in addition to affordable price warranty the mass production of the DM biosensors. The designed biosensors based on biocompatibility and stability will be safe for medical applications that contact body fluids. These properties are crucial for designing stable, cost-effective, efficient, and fast DM biosensors, which are vital in DM monitoring.

Here, we emphasized that using AuNSs accompanied by other nanomaterials and biological agents can improve the most influential aspects of successful DM biosensors, namely, sensitivity, specificity, assay time, and stability. This work covers AuNS applications in simple and easy-to use detection of many different DM biomarkers. Researchers are attempting to discover possible transformations to achieve stability and high sensitivity and selectivity. Substantial advances are expected. However, the approaches described in this review are based on experimental research that has as yet no significant commercialized application. Further consideration is required to translate these technologies into routine and functional biosensing devices. Another vital concern is that the existing glucose biosensors are not continuous, wearable, or implantable equipment. Developing non-invasive, continuous, and wearable biosensors can eliminate the discomfort associated with finger-prick tests, improving patient compliance and providing comprehensive DM management throughout the day. Meanwhile, challenges such as regulatory hurdles, scalability, cost, and user adaptation must be addressed. Another limitation of this work is the exclusion of the fourth-generation category of reagent-less glucose biosensors incorporating AuNSs. In conclusion, AuNSs, with their unique properties, can greatly improve the ability to detect DM biomarkers in biosensors. In this context, they are valuable nanomaterials for DM diagnosis, but further research and development are needed.
