# Corrigendum: Recent advances in gold nanostructure-based biosensors in detecting diabetes biomarkers

**DOI:** 10.3389/fbioe.2025.1526911

**Published:** 2025-02-19

**Authors:** Tahereh Jamshidnejad-Tosaramandani, Soheila Kashanian, Kobra Omidfar, Helgi B. Schiöth

**Affiliations:** ^1^ Nanobiotechnology Department, Faculty of Innovative Science and Technology, Razi University, Kermanshah, Iran; ^2^ Department of Surgical Sciences, Division of Functional Pharmacology and Neuroscience, Uppsala University, Uppsala, Sweden; ^3^ Biosensor Research Center, Endocrinology and Metabolism Molecular–Cellular Sciences Institute, Tehran University of Medical Sciences, Tehran, Iran; ^4^ Sensor and Biosensor Research Center (SBRC), Faculty of Chemistry, Razi University, Kermanshah, Iran; ^5^ Endocrinology and Metabolism Research Center, Endocrinology and Metabolism Clinical Sciences Institute, Tehran University of Medical Sciences, Tehran, Iran

**Keywords:** diabetes mellitus, biosensor, gold nanostructures, biomarker, glucose detection

In the published article, an author name was incorrectly written as **Helgi Schiöth**. The correct spelling is **Helgi B. Schiöth**.

The authors apologize for this error and state that this does not change the scientific conclusions of the article in any way. The original article has been updated.

